# Methamphetamine Alters the Normal Progression by Inducing Cell Cycle Arrest in Astrocytes

**DOI:** 10.1371/journal.pone.0109603

**Published:** 2014-10-07

**Authors:** Austin R. Jackson, Ankit Shah, Anil Kumar

**Affiliations:** Division of Pharmacology and Toxicology, University of Missouri-Kansas City School of Pharmacy, Kansas City, Missouri, United States of America; University of Nebraska Medical Center, United States of America

## Abstract

Methamphetamine (MA) is a potent psychostimulant with a high addictive capacity, which induces many deleterious effects on the brain. Chronic MA abuse leads to cognitive dysfunction and motor impairment. MA affects many cells in the brain, but the effects on astrocytes of repeated MA exposure is not well understood. In this report, we used Gene chip array to analyze the changes in the gene expression profile of primary human astrocytes treated with MA for 3 days. Range of genes were found to be differentially regulated, with a large number of genes significantly downregulated, including NEK2, TTK, TOP2A, and CCNE2. Gene ontology and pathway analysis showed a highly significant clustering of genes involved in cell cycle progression and DNA replication. Further pathway analysis showed that the genes downregulated by multiple MA treatment were critical for G2/M phase progression and G1/S transition. Cell cycle analysis of SVG astrocytes showed a significant reduction in the percentage of cell in the G2/M phase with a concomitant increase in G1 percentage. This was consistent with the gene array and validation data, which showed that repeated MA treatment downregulated the genes associated with cell cycle regulation. This is a novel finding, which explains the effect of MA treatment on astrocytes and has clear implication in neuroinflammation among the drug abusers.

## Introduction

Astrocytes are the most abundant cell type in the brain and are essential for neuronal survival and function. In addition, they contribute in formation and maintenance of the Blood Brain Barrier (BBB), serve as reservoirs for glycogen, and control ionic and osmotic homeostasis in the brain [1]. Beyond these functions, astrocytes also assist in the development of synapses as well as axon and dendrite outgrowth [2]. Apart from being an indispensable cell type of the brain, astrocytes are one of the innate immune responders in the brain. Particularly, astrocytes have been shown to activate immune responses against hantaviruses [3], toxoplasma [4], [5], and several bacterial agents [6]. However, repeated activation of astrocytes results in dysregulation of lipoxygenase and cyclooxygenase, leading to endothelial cell apoptosis [7]. Astrocytes are also highly affected by drugs of abuse, including methamphetamine (MA). Neurotoxic levels of MA results in reactive astrocytes that remain active up to 30 days [8]. This activation of astrocytes is partially dependent on sigma receptor and Signal Transducer and Activator of Transcription signaling, as shown by blockade with SN79, a sigma-receptor antagonist [9].

MA is a potent psychostimulant that promotes neuronal toxicity by several mechanisms such as release of monoamine neurotransmitters including dopamine, serotonin, and norephinephrine [10], induction of oxidative stress [11] and dysregulation of glucose uptake in neurons and astrocytes via Glucose transporter [12]. It is becoming increasingly evident that astrocytes play a critical role in MA-induced neuropathology [13]. MA abuse has been a pervasive problem; however, the precise underlying mechanism(s) of MA toxicity is unclear. Several studies have attempted to explain the effect of acute exposure to MA, while studies on repeated exposure are still scarce. MA is an acutely addictive substance meaning that one-time use is not common. Furthermore, repeated self-administration of MA can result in impaired attention, memory and executive function [14]. Moreover, repeated exposure to MA in rats causes distinct changes in the neurophysiology of the rat striatum including a sharp increase in oxidative stress and increased excitotoxicity [15]. Acute exposure to MA also results in oxidative stress that induces apoptosis through a cytochrome p450-mediated mechanism [16]. Furthermore, acute exposure of MA results in reactive astrocytes as measured by IL-6 and other proinflammatory cytokine induction [17], [18].

While many studies accurately reflect acute exposure to MA, very few studies exist that detail the effect of repeated MA exposure on astrocytes. To elucidate these effects, we used total transcriptome Gene Array to monitor changes in astrocytes that have been treated with MA for 3 days. The present study provides insight into MA abuse and the neurotoxicity associated with MA. Based on our transcriptome analysis, we further sought to validate functional impact of MA on cell cycle regulation.

## Materials and Methods

### Cells and Reagents

SVGA, an immortalized clone of SVG astrocytes, were cultured as previously described [16]. Primary astrocytes were isolated as previously described [16]. All use of primary astrocytes were approved by the UMKC IRB for use in our experiments. This study was determined to be non-human research because the samples are obtained from non-living subjects and was also approved by UMKC Institutional Biosafety Committee. Cells were maintained in DMEM supplemented with 10% FBS, 0.1% Gentamycin, Glutamine, and Non-Essential Amino Acids, sodium bicarbonate. Cells were cultured in a 37°C, 5% CO_2_ humidified incubator. MA was purchased from Sigma Alrdich (St. Louis, MO).

### MA Treatment

MA was added at a concentration of 500 µM for all experiments detailed in this study. This dose was decided based on previously reported blood concentrations and tissue/serum compartmentalization [19]–[21]. Primary astrocytes were treated with MA once a day for 3 days. For Cell cycle experiments, MA was added to SVGA in a T75 flask for 48 hours (once a day) followed by trypsinization and the cells were replated in 12 well plates with media containing MA, and cultured for a total duration of 72 hours. The cell cycle analysis was performed at various time points after 72-hour treatment with MA.

### Affymetrix 3′IVT Gene Array

RNA was extracted from cells either treated with MA or untreated control after 3 days using RNeasy isolation kit (Qiagen). RNA were spectrophotometrically analyzed to determine purity and diluted for the gene array. RNA was analyzed using Affymetrix Gene Chip expression analysis services provided by University of Kansas Medical Center (Kansas City, KS). Samples were analyzed on a 3′ IVT HumanU133A 2.0 gene chip. Data was analyzed using Affymetrix Expression Console and Transcriptome Analysis Software (TAC). The gene array was run in triplicate, and significance of the difference for each gene was determined by one-way ANOVA. Differentially regulated genes were defined as genes with a 2 fold or greater change over controls with a p<0.05.

### Gene Ontology

The Gene Array results were filtered on genes that were differentially regulated by 2 fold or greater. This gene list was used in Gene ontology analysis using the Database for Annotation, Visulaization, and Integrated Discovery (DAVID) and Cytoscape software. DAVID gene ontology databases were probed with Gene lists from upregulated or downregulated for Molecular Function, Cellular Compartment, and Biological processes using Cytoscape plugin ClueGO V2.1.1 using a term connection restriction cutoff of (kappa score) of 0.4.

### Pathway Analysis

DAVID gene ontology analysis gene lists were probed against KEGG reactome pathways to determine molecular pathways with high enrichment.

### Real Time Quantitative PCR

RNA was isolated from cells treated with 3 days of MA as detailed before. Quantitative RT-PCR was performed on those RNA samples as per manufacturer’s specifications using BioRad iScript One-Step kits. Shortly, 150 ng of RNA was added to each reaction and validated primers for each target gene were used at optimal temperature as determined by gradient PCR. Fold change was calculated for each sample using 2^−ΔΔCt^ method. Genes were normalized against expression of Hypoxanthine-guanine phosphoribosyl-transferase. The reaction consisted of reverse transcription at 50°C for 10 minutes followed by activation of polymerase at 95°C for 5 minutes. The amplification reaction was 10 seconds at 95°C followed by 30 seconds at the optimal T_m_ for each primer.

### Cell Cycle Analysis

The cells were treated as mentioned earlier under MA treatment section. After 48 hours of MA treatment, cells were counted by trypan blue exclusion and replated into 12 well plates at 2.5×10^5^ cells per well. A total of 72 hours after the initial MA treatment, cells were trypsinized every three hours. The cells were pelleted and washed twice with PBS. Cells were fixed in 66% ethanol for 24 hours before the start of the cell cycle analysis. After fixation, cells were washed three times with PBS and resuspended in 250 µl of FxCycle PI/RNase staining solution. The cells were then incubated at room temperature for 30 minutes followed by immediate analysis on FACSCanto II (BD Biosciences, San Jose, CA) flow cytometer. Cells were gated on live cells using forward and side scatter plots. Furthermore, cell aggregates were excluded by gating area vs. width peaks in the PE(505/585) channel. Cells were analyzed at 3, 6, 9 and 12 hours for progression through the cell cycle.

### Statistics

Data is expressed as means ± SE for aggregate data of at least three individual experiments. Each experiment was performed in triplicate. Statistical significance was calculated using one-way ANOVA and a value of p<0.05 was considered significant.

## Results

### Repeated MA Exposure Significantly Alters Gene Expression Profiles in Human Fetal Astrocytes

**Figure 1:**
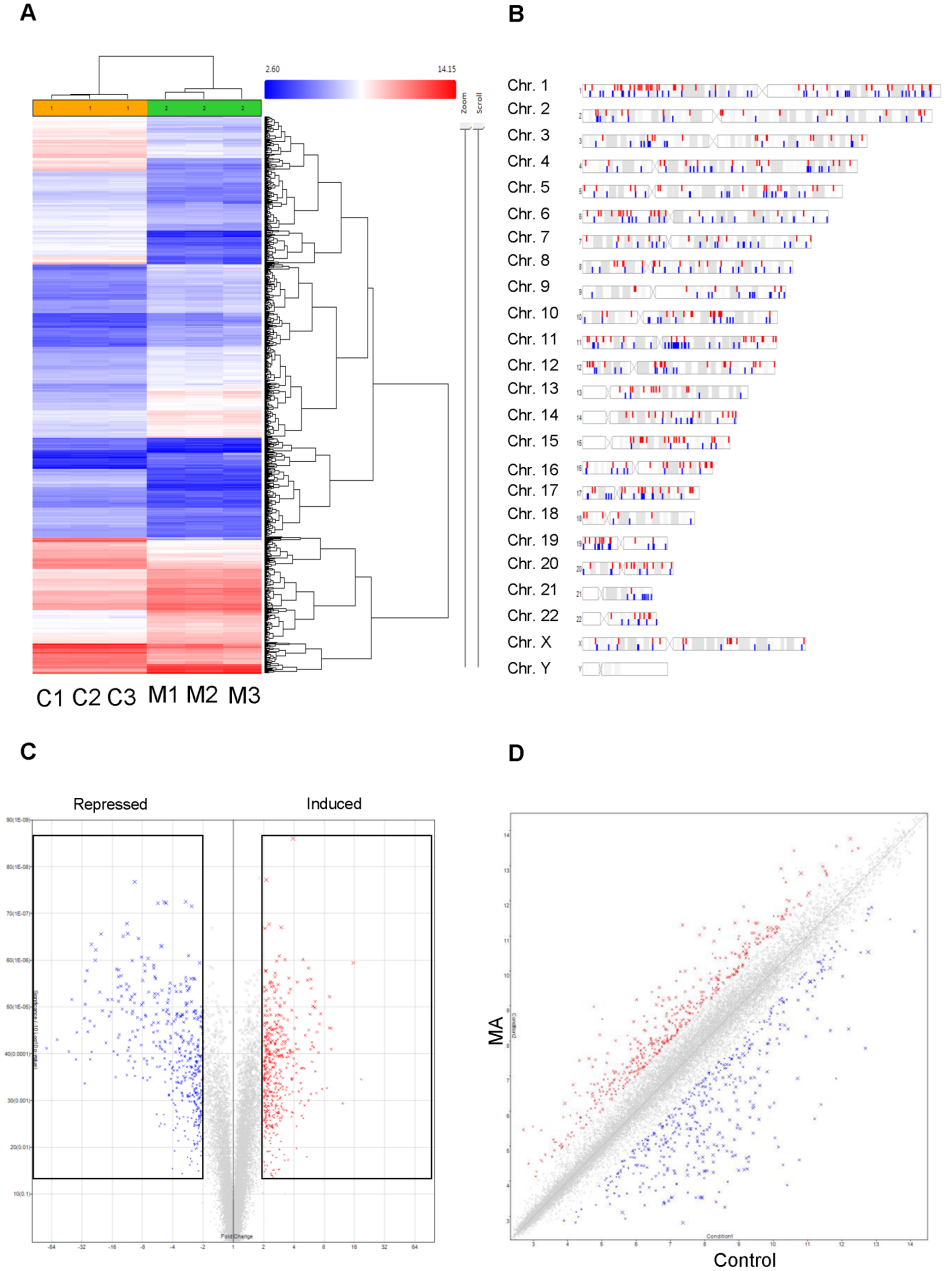
Repeated MA treatment causes significant changes in the gene expression profile in primary human astrocytes. Primary human astrocytes were isolated as noted before and treated once a day for 3 days with 500 µM MA. RNA was isolated and analyzed using Affymetrix 3′IVT Human gene chip. Analysis was performed using Affymetrix Expression console for normalization and Transcriptome Analysis Console (TAC) for annotation and fold change calculation. (A) Signal values were plotted and clustered in a heat map to determine overt differences in signal between the two samples. Clustering was performed by TAC. (B) Chromosome distribution analysis shows where the MA induced or repressed genes are located in the human genome. (C) Fold change was calculated by TAC software and displayed as a scatter plot. Genes upregulated by MA are shown in red and downregulated genes are shown in blue. (D) Fold change for each gene over 2.0 fold change was plotted against the significance as calculated by One-way ANOVA in a Volcano Plot. Genes within the black boxes for either induced or repressed expression were considered for further ontology and pathway analysis.

Human fetal astrocytes were exposed to 500 µM MA once a day. The cells were treated for three days at the same time each day to simulate repeated MA exposure. RNA was extracted from these cells and was analyzed using Affymetrix 3′IVT Gene Chip. The replicates for each treatment were very tightly grouped and showed very little variation as shown by a Pearson’s Correlation analysis (PCA) of the signal data, and the signal data showed a normal distribution (Data not shown). PCA analysis showed that the control group and the three-day MA treatment group were not similar and, in fact, showed highly different distribution against the principle components (Data not shown). We found many genes differentially regulated by MA treatment, but a cutoff of two fold or greater was used to selectively analyze genes that were altered by significantly higher margin when compared to untreated controls. After filtering, we found 777 genes downregulated and 696 genes upregulated by MA treatment. Fig. 1A shows a heat map distribution of those differentially regulated genes. The genes shown in red have high signal, while blue represents low signal. The heat map demonstrated that MA shows several areas with highly altered (up or down regulated) signals as compared to control. All analysis was performed using Transcriptome Analysis Console (Affymetrix). The genes were evenly distributed among the chromosomes, with no significant clustering on any one chromosome, which could suggest a site-specific gene induction (Fig. 1B). These genes are represented as a scatter plot (Fig. 1C) showing the upregulated genes in red and downregulated genes in blue. When these differentially regulated genes were plotted against significance (Fig. 1D), we found that the red dots from Fig. 1C represented significantly induced genes, while the blue dots from Fig. 1C represented significantly repressed genes. In addition, we found several genes that showed large magnitude (up or down regulated by at least two folds when compared to control) as well as high statistical significance (Fig. 1D). These figures show that MA significantly alters the expression profile of several genes in primary astrocytes after repeated administration for three days.

Table 1 shows the top 25 upregulated genes, with the highest upregulation being CXCL2 and CXCL10 with 46.14 and 28.0 fold, respectively. In the same way, Table 2 shows the top 25 downregulated genes, with the highest downregulation being −263.99 fold and −144.45 fold for MCM10 and RRM2, respectively. There were 102 genes that were downregulated below −10 fold, and 27 genes upregulated above 10 fold. This shows that MA is a very potent agent in changing gene expression.

**Table 1:** Top 25 genes induced by repeated MA treatment.

Fold Change	Gene Symbol	Gene Name
46.14	CXCL2	chemokine (C-X-C motif) ligand 2
28	CXCL10	chemokine (C-X-C motif) ligand 10
22.92	KRT14	keratin 14
18.39	PLA2G5	phospholipase A2, group V
18.15	RENBP	renin binding protein
18.07	ISG20	interferon stimulated exonuclease gene 20 kDa
17.31	AJAP1	adherens junctions associated protein 1
17.28	OGDHL	oxoglutarate dehydrogenase-like
16.83	FABP3	fatty acid binding protein 3, muscle and heart (mammary-derived growth inhibitor)
16.29	HRK	harakiri, BCL2 interacting protein (contains only BH3 domain)
15.73	PTGS2	prostaglandin-endoperoxide synthase 2 (prostaglandin G/H synthase and cyclooxygenase)
15.32	GAL	galanin/GMAP prepropeptide
14.57	GPNMB	glycoprotein (transmembrane) nmb
14.29	MVK	mevalonate kinase
14.09	LY96	lymphocyte antigen 96
13.97	CD177	CD177 molecule
13.89	DKK1	dickkopf WNT signaling pathway inhibitor 1
12.89	GDF15	growth differentiation factor 15
11.22	RASGRP3	RAS guanyl releasing protein 3 (calcium and DAG-regulated)
11.07	ANGPT2	angiopoietin 2
10.86	SLCO4C1	solute carrier organic anion transporter family, member 4C1
10.34	BAIAP3	BAI1-associated protein 3
10.19	THPO	thrombopoietin
10.08	ALDH1A2	aldehyde dehydrogenase 1 family, member A2
9.9	HMGA1	high mobility group AT-hook 1

**Table 2:** Top 25 Genes Repressed by Repeated MA treatment.

Fold change	Gene Symbol	Gene Name
−24.17	ABCC6	ATP-binding cassette, sub-family C (CFTR/MRP), member 6
−25.36	CCNB2	cyclin B2
−27.07	DTL	denticleless E3 ubiquitin protein ligase homolog (Drosophila)
−27.39	TOP2A	topoisomerase (DNA) II alpha 170 kDa
−27.91	RAD51AP1	RAD51 associated protein 1
−29.67	ASPM	asp (abnormal spindle) homolog, microcephaly associated (Drosophila)
−31.08	MKI67	antigen identified by monoclonal antibody Ki−67
−31.93	BIRC5	baculoviral IAP repeat containing 5
−32.17	BIRC5	baculoviral IAP repeat containing 5
−32.62	CEP55	centrosomal protein 55 kDa
−32.74	ACTC1	actin, alpha, cardiac muscle 1
−34.92	KIAA0101	KIAA0101
−34.99	ID1	inhibitor of DNA binding 1, dominant negative helix-loop-helix protein
−35.66	ORC1	origin recognition complex, subunit 1
−36.26	SHCBP1	SHC SH2-domain binding protein 1
−38.34	KIF20A	kinesin family member 20A
−42.22	TOP2A	topoisomerase (DNA) II alpha 170 kDa
−46.75	CDCA8	cell division cycle associated 8
−52.92	RRM2	ribonucleotide reductase M2
−65.95	PBK	PDZ binding kinase
−68.61	DLGAP5	discs, large (Drosophila) homolog-associated protein 5
−81.15	CDC45	cell division cycle 45
−81.74	KIFC1	kinesin family member C1
−104.35	HJURP	Holliday junction recognition protein
−144.45	RRM2	ribonucleotide reductase M2
−263.99	MCM10	minichromosome maintenance complex component 10

### Gene Ontology and Pathway Analysis of Gene Array Data

**Figure 2:**
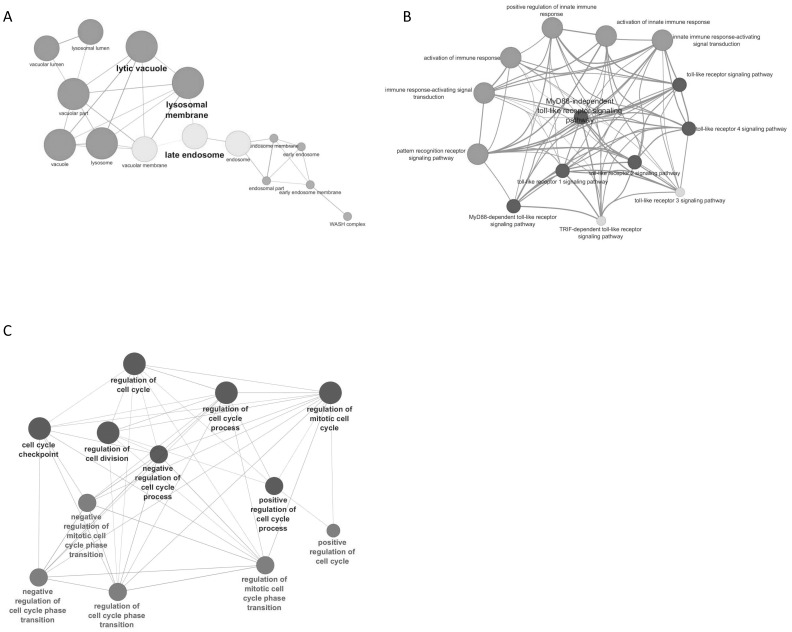
Gene ontology analysis of genes differentially regulated by repeated MA treatment. The gene list obtained from the TAC software was probed against gene ontology databases for Biologcial Pathway, Molecular Function, and Cellular compartment using Cytoscape software and the related plugin ClueGO. The highest ranked pathways were isolated and analyzed further. (A) Genes that were upregulated by MA treatment are found in a high proportion in the lysosome/endosome cellular compartment. (B) Gene upregulated by MA are involved in the activation of the innate immune response, with a high level of clustering in the Toll-like receptor pathway. (C) Genes downregulated by MA have a high clustering in the process of progression through the cell cycle and cell cycle regulation.

Using the Cytoscape plugin ClueGO, we performed gene ontology analysis on the genes that were differentially regulated by MA. We found that the genes that were upregulated by MA treatment fell into a few different categories when organized based on their cellular compartment. A large number of the genes upregulated by MA are localized to the lysosome/endosome pathway. Fig. 2A shows the relationship between these lysosome related genes. Many of the genes upregulated by MA are genes that are on the lysosomal membrane, while others are represented on the vacuole and endosomes. The genes found within these groups are included on Table 3. Within the lysosome itself, Lysosome-associated membrane protein 2 (LAMP2), DNA-damage regulated autophagy modulator 1 (DRAM1), and Capthesin A were found to be differentially regulated. This suggests some role in the regulation of the lysosome/endosome pathway. When probed for molecular function, we found that MA upregulated many genes involved in innate immunity. Since astrocytes are key immune responders in the brain therefore, alterations of these genes have implication in MA-mediated neuroinflammation. The genes upregulated by MA, including CXCL2, CXCL10, and LY96 are all involved in pattern recognition receptor signaling. Many of these genes upregulated by MA affected Toll-like receptor (TLR) signaling. Specifically, genes in TLR4, TLR3, TLR1, and TLR2 signaling pathways were significantly upregulated (Fig. 2B). These genes are also involved in the activation of the innate immune response through MyD88-dependent and MyD88-independent signaling [22]. Interestingly, MA treatment also upregulated the levels of Jun and Fos (Table 4), the subunits which make up Activating Protein-1 [23]. This data suggests that MA is involved in modulating the innate immune responses in astrocytes (Table 4). Finally, majority of the downregulated genes in MA treated cells belonged to cell cycle regulation pathway (Table 5). Fig. 2C shows that several categories of genes involved in cell cycle regulations were downregulated by MA. Included in these lists are genes that affect transition between cell cycle phases, regulation of mitotic cell phase initiation, genes that both positively and negatively regulate the cell cycle, and genes involved in cell cycle checkpoints. Table 5 shows the genes that are affected by MA treatment that fall into these different categories. This functional category of genes was by far the most significant of any functions affected by MA treatment. The genes included in table 5 are also known to affect DNA replication and monitor DNA integrity Particularly, DNA topoisomerase 2-alpha (TOP2A), dual specificity protein kinase TTK (TTK), and never in mitosis gene a-related kinase 2 (NEK2) were found to be significantly altered due to MA treatment [24]–[26].

**Table 3:** Genes induced by repeated MA treatment found in the Lysosome compartment.

WASH Complex	[FAM21A, FAM21B, FAM21C]
Endosomalpart	[CD1D, CD63, CLCN6, EPHB1, FAM21A, FAM21B, FAM21C, HLA-DQB1, KIF16B, LAMP2, LDLR, NPC1, NTRK2, PRLR, SLC11A2, STEAP1, STEAP3, TF, TFRC]
Endosomalmembrane	[CD1D, CD63, CLCN6, EPHB1, FAM21A, FAM21B, FAM21C, HLA-DQB1, KIF16B, LAMP2, LDLR, NPC1, NTRK2, SLC11A2, STEAP1, STEAP3, TF, TFRC]
EarlyEndosome	[APOE, CD8B, CLN3, CTNS, EPHB1, FAM21A, FAM21B, FAM21C, KIF16B, LDLR, SLC11A2, TF]
Vacuolarmembrane	[AP1S2, CD1D, CD63, CLN3, CTNS, DRAM1, GBA, HLA-DQB1, IGF2R, LAMP2, M6PR, MAP1LC3C, NEU1, NPC1, SLC11A2, SLC17A5, SLC36A1]
Lysosome	[ADA, AP1S2, CD1D, CD63, CLN3, CST3, CTNS, CTSA, CTSC, DNASE2, DRAM1, FYCO1, GBA, GLB1, GM2A, GNS, HEXA, HEXB, HLA-DQB1, HPS1, IDS, IGF2R, LAMP2, LDLR, LIPA, M6PR, MANBA, NAGLU, NEU1, NPC1, NPC2, RRAGD, SLC11A2,
LysosomalLumen	[CTSA, GBA, GLB1, GM2A, HEXA, HEXB, NEU1, TPP1]
Lyticvacuole	[ADA, AP1S2, CD1D, CD63, CLN3, CST3, CTNS, CTSA, CTSC, DNASE2, DRAM1, FYCO1, GBA, GLB1, GM2A, GNS, HEXA, HEXB, HLA-DQB1, HPS1, IDS, IGF2R,LAMP2, LDLR, LIPA, M6PR, MANBA, NAGLU, NEU1, NPC1, NPC2, RRAGD, SLC11A2,
LateEndosome	[APOE, BST2, CD63, CLN3, CST3, CTNS, FYCO1, LAMP2, M6PR, NPC1, PIK3R4, SLC11A2, SLC2A4, SQSTM1, STEAP3, TF]
Endosome	[APOE, BACE2, BST2, CD1D, CD63, CD8B, CDH1, CLCN6, CLN3, CST3, CTNS, EPHB1, EXPH5, FAM21A, FAM21B, FAM21C, FLT1, FYCO1, HLA-DQB1, IGF2R,KIF16B, LAMP2, LDLR, M6PR, NPC1, NTRK2, PIK3R4, PRLR, SLC11A2, SLC2A4, SQSTM1,

**Table 4:** Genes induced by repeated MA treatment have function in the innate immune reponse.

Activation of the immune system	[ADA, CFB, CXCL10, DUSP3, DUSP4, DUSP6, FOS, HLA-DQB1, IFIH1, JUN, LY96, LYN, MBL2, MICA, NR1D1, NR1H3, PRKCB, TLR1, TRAT1]
Signal Transduction	[ADA, DUSP3, DUSP4, DUSP6, FOS, HLA-DQB1, IFIH1, JUN, LY96, LYN, MICA, NR1D1, NR1H3, PRKCB, TLR1, TRAT1]
PRR signalling	[DUSP3, DUSP4, DUSP6, FOS, IFIH1, JUN, LY96, LYN, NR1D1, NR1H3, TLR1]
Positive regulation of Innate Immunity	[CD1D, DUSP3, DUSP4, DUSP6, FOS, IFIH1, JUN, LY96, LYN, MICA, NR1D1, NR1H3, SH2D1A, TLR1]
Activation of the innate immune system	[DUSP3, DUSP4, DUSP6, FOS, IFIH1, JUN, LY96, LYN, MICA, NR1D1, NR1H3, TLR1]
TLR1	[DUSP3, DUSP4, DUSP6, FOS, JUN, LY96]
TLR3	[DUSP3, DUSP4, DUSP6, FOS, JUN]
TLR2	[DUSP3, DUSP4, DUSP6, FOS, JUN, LY96, LYN]
TLR4	[DUSP3, DUSP4, DUSP6, FOS, JUN, LY96, LYN, NR1D1, NR1H3]

**Table 5:** Genes Repressed by Repeated MA treatment have function in the regulation of the Cell Cycle.

Regulation of Cell Cycle	[ASCL1, AURKA, AURKB, BIRC5, BLM, BORA, BRCA1, BRCA2, BUB1, BUB1B, CCNA2, CCNB1, CCNB2, CCNE2, CCNF, CDC20, CDC25A, CDC25C, CDC6, CDK1, CDK2, CDKN2C, CDT1, CENPE, CENPF, CENPJ, CHEK1, CKS1B, CKS2, DLGAP5, DTL, E2F1, E2F8, ECT2, EDN1, ESPL1, EZH2, FBXO5, FGFR3, FOXM1, GADD45G, GMNN, GTSE1, ID2, ID3, INSM1, KHDRBS1, KIAA0101, KIF20B, KIF23, KLHL22, KNTC1, LEF1, MAD2L1, MCM2, MLF1, MSX1, MSX2, NEK2, NGF, NKX3-1, NUSAP1, PDGFB, PKMYT1, PLK1, PLK4, PRKCA, RACGAP1, RBL1, RFWD3, RGCC, RNASEH2A, SKP2, TACC3, THBS1, TPX2, TTK, UBE2C, USP2, USP22, ZWILCH, ZWINT]
Regulation of Cell Division	[ASPM, AURKA, AURKB, BIRC5, BLM, BORA, BRCA2, BUB1, BUB1B, CDC20, CDC25C, CDC6, CENPE, CENPF, CHEK1, DLGAP5, E2F8, ECT2, EDN1, ESPL1, FBXO5, FGF4, FGFR3, KIF18B, KIF20B, KIF23, KLHL22, KNTC1, MAD2L1, NEK2, NKX3-1, NUSAP1, PDGFB, PKMYT1, PLK1, RACGAP1, RGCC, TGFB3, TTK, UBE2C]
Regulation of Cell Cycle process	[AURKA, AURKB, BIRC5, BORA, BRCA1, BRCA2, BUB1, BUB1B, CCNA2, CCNB1, CCNF, CDC20, CDC25C, CDC6, CDK1, CDK2, CDT1, CENPE, CENPF, CENPJ, CHEK1, DLGAP5, E2F1, E2F8, ECT2, EDN1, ESPL1, EZH2, FBXO5, FGFR3, FOXM1, GTSE1, ID2, INSM1, KIF20B, KIF23, KLHL22, KNTC1, LEF1, MAD2L1, MSX1, MSX2, NEK2, NUSAP1, PDGFB, PKMYT1, PLK1, PLK4, PRKCA, RACGAP1, RFWD3, RGCC, TPX2, TTK, UBE2C, ZWILCH, ZWINT]
Regulation of mitotic cell cycle	[AURKA, BIRC5, BORA, BRCA2, BUB1, BUB1B, CCNA2, CCNB1, CDC20, CDC25C, CDC6, CDK1, CDK2, CENPE, CENPF, CHEK1, DLGAP5, E2F1, EDN1, ESPL1, EZH2, FBXO5, FGFR3, GTSE1, ID2, KIF20B, KLHL22, KNTC1, MAD2L1, NEK2, NKX3-1, NUSAP1, PDGFB, PKMYT1, PLK1, PRKCA, RFWD3, RGCC, TPX2, TTK, UBE2C, USP2, USP22, ZWILCH, ZWINT]
Cell Cycle Checkpoint	[ASCL1, AURKA, AURKB, BIRC5, BLM, BORA, BRCA1, BRCA2, BUB1, BUB1B, CCNA2, CCNB1, CCNB2, CCNE2, CCNF, CDC20, CDC25A, CDC25C, CDC6, CDK1, CDK2, CDKN2C, CDT1, CENPE, CENPF, CENPJ, CHEK1, CKS1B, CKS2, DLGAP5, DTL, E2F1, E2F8, ECT2, EDN1, ESPL1, EZH2, FBXO5, FGFR3, FOXM1, GADD45G, GMNN, GTSE1, ID2, ID3, INSM1, KHDRBS1, KIAA0101, KIF20B, KIF23, KLHL22, KNTC1, LEF1, MAD2L1, MCM2, MLF1, MSX1, MSX2, NEK2, NGF, NKX3-1, NUSAP1, PDGFB, PKMYT1, PLK1, PLK4, PRKCA, RACGAP1, RBL1, RFWD3, RGCC, RNASEH2A, SKP2, TACC3, THBS1, TPX2, TTK, UBE2C, USP2, USP22, ZWILCH, ZWINT]

After functional annotation, we used the DAVID program to further characterize the genes affected by MA treatment. We found that when we performed gene functional classification on genes that were downregulated by MA treatment, the gene group with the highest enrichment score included many genes that affected the cell cycle and progression through mitosis. Of the 136 genes in the highest ranked cluster, 90 genes were found to be involved in the cell cycle, with 65 of those genes being important for M phase or transition into M phase. Furthermore, the gene clustering score, a measure of the relatedness of these genes, provided us with a keen insight into how repeated MA could be affecting cellular division.

### Pathway Analysis

**Figure 3:**
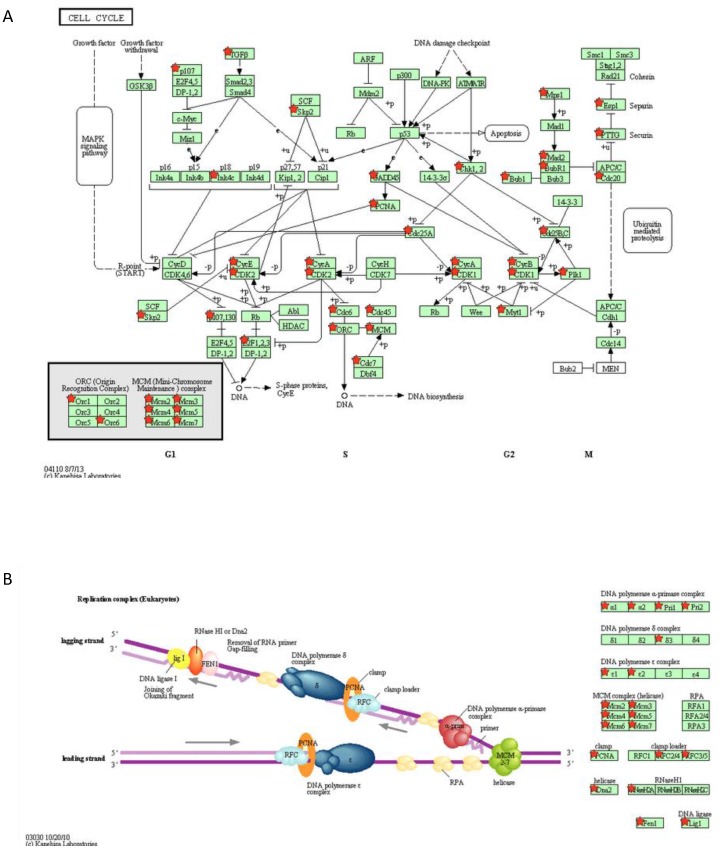
Pathway analysis of genes downregulated by repeated MA treatment shows effect in cell cycle progression. Using DAVID, the genes that were downregulated by MA treatment were used to determine the importance for those genes in biological pathways. (A) The 90 genes that were found to be involved the cell cycle showed effects in the transition between phases of the cell cycle. Several of the cyclin genes (CycE, A, B) were downregulated as well as several subunits of the Mini-Chromosome Maintenance Complex (MCM). (B) DNA replication pathway was also affected by repeated MA treatment. The DNA polymerase complex as well as the DNA clamp had several subunits downregulated by MA treatment.

Using KEGG pathway maps, we were able to determine the effect of repeated MA treatment on the progression through the cell cycle. We found that several genes involved in the regulation of cell cycle were affected by MA treatment. However, the overall impact of the altered genes on cell cycle was unclear. Fig. 3A shows the pathway map for cell cycle analysis, with the red stars representing genes that were downregulated by repeated MA treatment. In particular, all six subunits of the mini-chromosome maintenance complex were found to be downregulated by MA treatment. Similarly, two of the origin recognition complex (ORC) subunits were also found to be downregulated. Furthermore, several of the cyclins and cyclin dependent kinases (CDK), which are important for proper transition through the cell cycle, were also downregulated by more than two folds. Particularly, Cyclin E2 (−16.05 fold), Cyclin B2 (−25.36 fold), Cyclin F (−2.27), Cyclin A2 (−5.88 fold), and Cyclin B1 (−8.55 fold) were all downregulated by repeated MA treatment. Along with these, CDK2 (−2.24 fold) and CDK1 (−6.66 fold) were also found to be downregulated by repeated MA treatment. This suggests a significant role for repeated MA treatment in disrupting the normal cell cycle. Interestingly, MA treatment also upregulated the expression of growth arrest-specific 1 (2.52 Fold) which has been shown to disrupt normal cellular proliferation [27].

Along with cell cycle control, pathway analysis shows that several of the key regulators for DNA replication are downregulated by repeated MA administration. Fig. 3B shows the KEGG pathway for eukaryotic DNA replication. Altogether, the results clearly demonstrate that repeated MA administration affects several genes involved in the regulation of DNA replication process. Of note, TOP2A (−27.39 Fold), several units of the DNA polymerase holoenzyme, and DNA ligase (−3.55 fold) were all found to be downregulated by MA treatment. The preponderance of genes involved in the progression through the cell cycle, critical checkpoint genes, and genes that are responsible for proper DNA replication downregulated by MA treatment strongly supports a role for repeated MA treatment in the disruption of proper cellular proliferation and progression through the cell cycle.

### Validation of Gene Array Data

**Figure 4:**
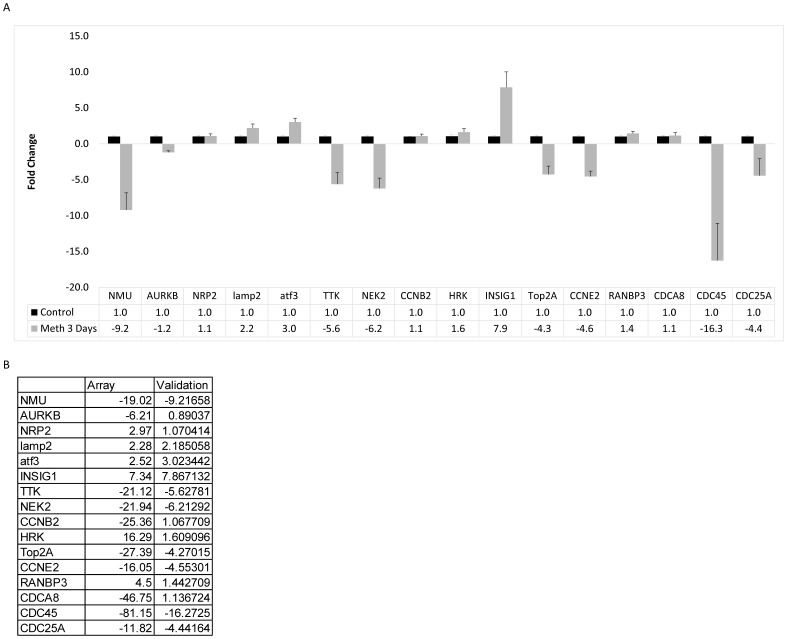
qPCR validation of Gene Array data. Primary astrocytes were treated with MA for 3 days and RNA was analyzed for genes affected by MA treatment as shown by the gene array. (A) Genes that were both upregulated and downregulated as seen in the gene array were chosen to validate the array data. Of the 16 genes chosen, 10 genes validated the array results, with 7 genes downregulated and 3 genes upregulated. (B) Comparison of the fold change results from the gene array and the qPCR validation.

To validate the changes in the genes that were observed in whole transcriptome gene chip, we used human fetal astrocytes from at least three different donors. The primary astrocytes were treated with MA as before and the RNA expressions were measured using real time RT-PCR. As shown in Fig. 4A, our validation confirmed several of the genes from the cell cycle pathway that were altered in gene array. Based on our analyses performed with the gene ontology and the functional annotation, cell cycle regulation pathway scored the highest among all therefore, we chose to validate the genes involved in regulation of the cell cycle that were found to be highly downregulated by MA treatment in our gene array. Therefore, we validated various genes involved in the cell cycle regulation such as TTK, NEK2, cyclin B2 (CCNB2), TOP2A, cyclin E2 (CCNE2), cell division cycle associated 8 (CDCA8), cell division cycle 45 (CDC45), and cell division cycle 25A (CDC25A) [28]–[30]. All of these genes except CCNB2 and CDCA8 confirmed expression profiles as observed in the gene array (Fig. 4). However, the expression levels measured in RT-PCR were different when compared to those in gene array analysis. This is not surprising since PCR is more sensitive of an assay than whole gene chip array and the difference in signals between samples can be better resolved. Several other genes that we chose in an attempt to validate the array data also showed the same effect as seen in the gene chip. Particularly, validation data for Neuromedin U (NMU), Cyclic AMP-dependent transcription factor 3 (ATF3), Insulin-induced gene 1 (INSIG1), and LAMP2 showed the levels of expression that were very close to that observed in the gene chip array (Fig. 4A–4B). Based on the validation data for cell cycle related genes, we concluded that the repeated MA treatment indeed disrupted the normal homeostasis of these genes.

### Repeated MA Treatment Disrupts Normal Progression through the Cell Cycle

**Figure 5:**
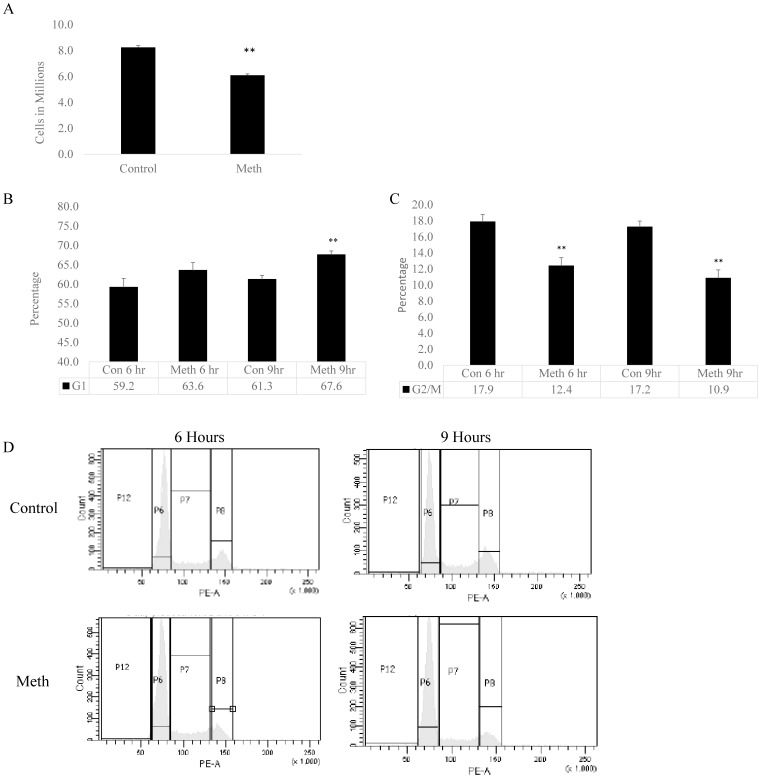
Repeated MA treatment alters the normal progression of astrocytes through the cell cycle. SVG Astrocytes were treated once a day with 500 µM and analyzed for proliferation and progression through the cell cycle. (A) Cells were plated in a T75 and treated with MA for 2 days and counted using Trypan blue exlusion. (B–D) Cells were treated with MA for 3 days. After 3 days of treatment, SVGA cells were analyzed for cell cycle status at 6 and 9 hours after the last treatment. The statistical significance was calculated in one way ANOVA and ** denotes significance of <0.001.

As seen in the gene array and the validation, when astrocytes are repeatedly treated with MA, several important cell cycle related genes are differentially regulated. Therefore it was imperative to believe that MA would affect the cell cycle. To test this, we treated SVGA cells with 500 µM MA as before and monitored their proliferation. Cells were seeded in a T75 flask at 2×10^6^ cells per flask and treated for two days with MA. At the end of the two-day culture, we counted viable cells using trypan blue exclusion. We found that flasks treated with MA had significantly fewer cells than untreated control cells (Fig. 5A), which suggested that MA affected the proliferation of these cells. To confirm the effect of MA on cell cycle progression, we assessed the cells undergoing various stages of the cell cycle using PI staining. We monitored the cells every three hours after the three-day treatment with MA. Fig. 5 shows that control cells were 59.23% in G1 (Fig. 5B) and 17.87% in G2 phase (Fig. 5C) at 6 hours. However, MA treated cells showed a higher G1 percentage (63.58%) and a reduced G2/M percentage (12.38%). This shows that cells treated with MA were not progressing through the cell cycle as control cells were. To confirm this, we also measured the cell cycle progression at 9 hours. We saw a similar response to what we saw at 6 hours. However, the response was exaggerated. Control cells showed 61.26% and 17.22% G1 and G2/M, respectively. This was not overtly different than what was found at 6 hours. However, MA treatment showed a further increased G1 phase peak (67.60%) with a further depressed G2/M peak (10.86%). This showed that cells were not transitioning from S phase to G2/M phase at the same level in MA treated samples than they were in control treated samples. It is noteworthy that the proportion of cells undergoing the S phase was same for both, control and MA treated samples at 6 and 9 hours. However, there was marginal (∼2%) increase (no statistical significance) in S phase staining cells in MA treated samples at 6 hours. Fig. 5D shows representative flow cytometry histograms. As seen, the control cells show greater G2/M peak as shown by the P8 gate that is depressed in MA treated cells. This shows that MA negatively affects the progression of cells through the cell cycle and the proliferation of these cells as an end result.

## Discussion

A variety of illicit drugs including MA have been shown to exhibit neurotoxic potential[31]–[33]. The neurotoxicity associated with MA is attributed to increased oxidative stress, dopamine dysfunction, mitochondrial dysfunction, excitotoxicity, alteration in BBB integrity, inflammatory mechanisms and defective neurogenesis [31], [33]. In particular, MA has been shown to inhibit neurogenesis in subventricular and hippocampal progenitor cells [34], [35]. In addition, classical fMRI study on MA demonstrated reduced striatal volumes in children with prenatal MA exposure. This study also showed reduced density of dopamine transporters (DAT) and D2 receptors in MA abusing individuals [36]. More recently, MA has also been shown to decrease immature hippocampal neurons by reducing progenitor cells. This was shown to reduce a total pool of S-phase progenitor cells without affecting the s-phase dynamics [37]. In this study, we sought to understand the mechanisms of neurotoxicity induced by repeated MA treatment by using 3′IVT transcriptome gene array analysis. An earlier genome-wide study showed MA-mediated changes in cell cycle genes in rat striatum [38]. However, till date no study has shown the transcriptome wide effect of multiple treatments of MA on primary human astrocytes. Thus, the present study provides important findings toward understanding the neurotoxic mechanisms of MA treatment in astrocytes. Our transcriptome-wide array showed significant alterations in the expression profile of various genes in the astrocytes as seen by the large number of genes differentially regulated by repeated treatments of MA (Fig. 1). MA has been shown to be neurotoxic after acute treatment by a number of mechanisms [12], [16], [39] but it remains unclear how MA affects astrocytes after repeated exposure. The present study shows that once a day treatment for 3 days drastically changes the gene expression profile, with 1473 differentially regulated genes (777 downregulated and 696 upregulated genes) by greater than 2 fold. Gene ontology analysis showed several different pathways affected by repeated MA exposure, but the most significantly affected pathway was in the regulation of progression through the cell cycle (Fig. 2). Upon further examination, we found that many genes that are important for the regulation of proper progression through the cell cycle showed up as downregulated by repeated MA exposure, and pathway analysis showed that majority of these altered genes belonged to the Cyclin and CDK family pathways (Fig. 3). Other genes were found to be downregulated when we performed the cell cycle pathway analysis, including TOP2A, NEK, and TTK, which are important for mitosis and maintaining DNA integrity [40]–[42]. Based on this analysis, we wished to validate the expression of the genes critical for cell cycle progression. Using qPCR, we validated the expression of several genes that were shown in the gene array, which further confirmed the results obtained with the gene array (Fig. 4). Since several of the cell cycle related genes were validated in qPCR, we studied the effect of repeated MA treatment on disruption of the normal cell cycle progression. We observed a reduction in G2/M progression in MA treated samples at two different time points (6 hours and 9 hours) when compared with their respective controls (Fig. 5). The results suggested that MA affects the cell cycle in astrocytes and their ability to proliferate.

Earlier findings from animal studies have shown that repeated MA exposure negatively affects the overall behavior and motor functions [43], [44]. Although several studies involving astrocyte treatment with MA focus on short-term exposure [8], [13], [39], [45], very few studies have been attempted to examine the effect of repeated exposure of MA to astrocytes. Our present study uses a multiple exposure approach using primary astrocytes exposed to repeated doses of MA over the course of three days. The results in the present study provide an insight into the molecular mechanism of astrocytic cell death in chronic MA use and the possible neurotoxic mechanisms. MA has been shown to affect the ability of astrocytes to perform key functions [12], [46], [47] and leads to reactive astrogliosis [9], [48]. Along with these effects, acute treatment of MA upregulates cytokines in astrocytes [18], [49] as well as other cells, including macrophages [50]. Our data in the gene array are consistent with these earlier reports and shows that many of the upregulated genes were involved in the activation of the innate immune system, including very high expression of CXCL2 and CXCL10. Astrocytes are key inflammatory cells in the brain, and any alteration of their expression profile would lead to an imbalance in the inflammatory response in the brain which has been shown to be deleterious when improperly controlled [51]–[53]. The altered expressions of cytokines and chemokines in gene array also further confirm the role of MA in neuroinflammation. However, detailed mechanism(s) underlying the MA-mediated cytokine/chemokine production requires more rigorous studies. Also, the biological outcome of MA-mediated upregulation of chemokines in astrocytes is unclear and further study is needed to elucidate the significance of these upregulated genes to the brain.

A variety of cell cycle genes that were found to be downregulated in our study as shown in Fig. 4 (NEK2, TTK, CDCA8, CDC25, TOP2A, and CCNE2), have also been reported to be clinically important [28]–[30]. For example NEK2 inhibitors have been developed as possible chemotherapeutic interventions because NEK2 is overexpressed in several different cancer, including breast [54] and ovarian cancers [55]. It is critical for maintaining genomic stability during mitosis, so any loss of NEK2 function would result in dysfunctional mitotic division and thus inhibited proliferation [41]. TTK, also called human protein kinase monopolar spindle 1 (MPS1), is a critical mitotic checkpoint protein. TTK phosphorylates CHK2, thus activating the mitotic checkpoint and allowing for passage through the cell cycle [56]. TOP2A, in a similar way to NEK2 and TTK2, is an important gene in the regulation of G2/M progression [42]. CCNE2 is a canonical cell cycle transition protein, with an important role in the passage from G1 to S phase [57]. All these genes taken together explains our findings with the cell cycle analysis. We observed fewer G2/M phase cells at 6 and 9 hours after three-day MA administration with a concomitant G1 phase increase. This suggests that MA-treated astrocytes are unable to pass into the G2/M phase or transition out of the G1 phase. In summary, the present study provides evidence that MA affects the transition through the cell cycle. Further studies to ascertain complete molecular mechanisms will provide essential information in the process such as astrogliosis, which is commonly observed in several neurological disorders as a result of neuroinflammation [58].

In conclusion, the present study is the first report showing transcriptome-wide gene expression due to repeated MA-treatment in astrocytes. Our gene array analysis provides several pathways that have functional implications in cell cycle regulation, immune function and DNA replication. Understanding the precise molecular mechanism(s) associated with these functional outcomes due to repeated MA use is of critical importance to find a root cause for MA-mediated neurotoxicity. The neurotoxicity associated with MA is multifaceted and MA exhibits varied effects on different cell types [11], [15], [18], [47], [59]. Understanding the cellular biology in response to MA administration allows for better treatment interventions and gives new targets for pharmaceutical development. In particular, the ability of MA to alter the astrocyte proliferation underscores its role in neuroinflammation and astrogliosis. The present study provides a new direction in MA-associated neurotoxicity, which has direct clinical implication for MA abusers.
